# Clinical Pathophysiology of Human T-Lymphotropic Virus-Type 1-Associated Myelopathy/Tropical Spastic Paraparesis

**DOI:** 10.3389/fmicb.2012.00389

**Published:** 2012-11-09

**Authors:** Yoshihisa Yamano, Tomoo Sato

**Affiliations:** ^1^Department of Rare Diseases Research, Institute of Medical Science, St. Marianna University School of MedicineKawasaki, Japan

**Keywords:** epidemiology, diagnosis, HAM/TSP, HTLV-1, pathogenesis, prognosis, retrovirus, treatment

## Abstract

Human T-lymphotropic virus type 1 (HTLV-1), a human retrovirus, is the causative agent of a progressive neurological disease termed HTLV-1-associated myelopathy/tropical spastic paraparesis (HAM/TSP). HAM/TSP is a chronic inflammatory disease of the central nervous system and is characterized by unremitting myelopathic symptoms such as spastic paraparesis, lower limb sensory disturbance, and bladder/bowel dysfunction. Approximately 0.25–3.8% of HTLV-1-infected individuals develop HAM/TSP, which is more common in women than in men. Since the discovery of HAM/TSP, significant advances have been made with respect to elucidating the virological, molecular, and immunopathological mechanisms underlying this disease. These findings suggest that spinal cord invasion by HTLV-1-infected T cells triggers a strong virus-specific immune response and increases proinflammatory cytokine and chemokine production, leading to chronic lymphocytic inflammation and tissue damage in spinal cord lesions. However, little progress has been made in the development of an optimal treatment for HAM/TSP, more specifically in the identification of biomarkers for predicting disease progression and of molecular targets for novel therapeutic strategies targeting the underlying pathological mechanisms. This review summarizes current clinical and pathophysiological knowledge on HAM/TSP and discusses future focus areas for research on this disease.

## Epidemiology

Human T-lymphotropic virus type 1 (HTLV-1), the first human retrovirus discovered (Poiesz et al., 1980), infects approximately 10–20 million people worldwide (de Thé and Bomford, 1993). Endemic areas of HTLV-1 infection include the Caribbean, southern Japan, Central and South America, the Middle East, Melanesia, and equatorial Africa (Blattner and Gallo, 1985; Gessain and de Thé, 1996). Although majority of the infected individuals remain lifelong asymptomatic carriers, approximately 0.25–3.8% develop a progressive neurological disease termed HTLV-1-associated myelopathy/tropical spastic paraparesis (HAM/TSP; de Thé et al., 1985; Osame et al., 1986a) and 2–5% develop an aggressive mature T cell malignancy termed adult T cell leukemia/lymphoma (ATLL; Uchiyama et al., 1977; Hinuma et al., 1981). HAM/TSP is two to three times more common in women than men. In a prospective cohort analysis, the onset period after infection ranged from 4 months to 30 years (median, 3.3 years; Maloney et al., 1998). HTLV-1 is primarily transmitted by breast feeding, but also spread via sexual intercourse, blood transfusion, and sharing of needles. While ATLL is mainly associated with breast feeding, HAM/TSP can be occurred in infected individuals of any route of transmission (Sugiyama et al., 1986; Tajima et al., 1987; Osame et al., 1990a; Krämer et al., 1995; Maloney et al., 1998). In Japan, nationwide routine screening of the anti-HTLV-1 antibody for blood donations is conducted after the high incidence of HAM/TSP in recipients of blood transfusion reported in 1986 (Osame et al., 1986b) and such screening has proven to be an effective way of curbing transfusion-related infection (Kamihira et al., 1987). In Japan, the lifetime risk of developing HAM/TSP among approximately one million HTLV-1-infected individuals is 0.25% (Kaplan et al., 1990). The lifetime risk of HAM/TSP in the estimated 22,000 HTLV-1-infected individuals in England is 3% (Tosswill et al., 2000). Seroprevalence of HTLV-I in blood donors in the United States is 1 per 10,000 individuals. A recent study estimates that approximately 266,000 individuals are infected with HTLV-1 or 2, and that there are likely more than 3600 people in the United States with unrecognized HAM/TSP (Orland et al., 2003).

## Clinical Features

HTLV-1-associated myelopathy/tropical spastic paraparesis mainly presents as a slowly progressive spastic paraparesis with neurogenic bladder disturbance (Nakagawa et al., 1995; Araújo et al., 1998). The first major symptoms are typically gait disturbance, tendency to fall, stumbling, leg weakness, back pain, bladder/bowel, and sexual dysfunction, which are usually insidious but occasionally occur abruptly over weeks. Symptoms in the lower limbs are mostly symmetrical. Neurogenic bladder symptoms such as urinary frequency, urgency, incontinence, and/or retention are very common and seen very early in the course of the disease; sometimes, these symptoms precede the development of paraparesis by many years. The patients have a spastic gait with weakness of the lower limbs, which is most evident proximally. Hyperreflexia of the lower limbs is commonly seen, often accompanied by clonus and Babinski’s sign, and hyperreflexia of upper limbs is occasionally observed in some patients. Upper limb power is usually retained throughout the course of the disease. Sensory disturbance – typically paresthesia of the feet and occasionally of the hands – is observed in some HAM/TSP patients and is generally mild. Sensory level is occasionally observed at the lower thoracic spinal cord, although a clear-cut sensory level is unusual. Loss of light touch sensation and pain in the lower limbs were reported in 27–53% of patients in three clinical series, with impairment of vibration sense recorded in 3–48% of the patients (Vernant et al., 1987; Bhigjee et al., 1990; Araújo et al., 1993). Pain and numbness, usually at the lumbar level and lower limbs, is present in approximately 5–50% of the patients (Gotuzzo et al., 2004). In some cases, pain is severe and more distressing than gait disturbance. Back pain, constipation, and sexual dysfunction are also very common (Verdonck et al., 2007). The less common signs and symptoms include cerebellar signs, optic neuritis and atrophy, and nystagmus (Table 1).

**Table 1:** **Clinical features of HAM/TSP**.

**Motor Disturbance**
Symptoms: gait disturbance, tendency to fall, stumbling, and leg weakness
Signs: spastic paraparesis, weakness and hyperreflexia of the lower limbs, clonus, and Babinski’s sign
**Sensory Disturbance**
Symptoms: pain and numbness at the lumbar level and lower limbs and back pain
Signs: paresthesia of the feet and occasionally of the hands, sensory level at the lower thoracic spinal cord, loss of light touch sensation
**Autonomic Dysfunction**
Symptoms: urinary frequency, urgency, incontinence, retention, constipation, and sexual dysfunction
Signs: neurogenic bladder, overactive bladder, diminished peristalsis, and erectile dysfunction

Human T-lymphotropic virus type 1 is also associated with non-neoplastic inflammatory conditions such as HTLV-1-associated uveitis (Mochizuki, 1992), Sjögren syndrome (Eguchi et al., 1992), bronchoalveolitis (Nakagawa et al., 1995), arthritis (Nishioka et al., 1989), and polymyositis (Morgan et al., 1989), in which high tissue concentrations of HTLV-1-infected T lymphocytes have been observed. Importantly, some HAM/TSP patients have more than one of these HTLV-1-associated inflammatory conditions (Nakagawa et al., 1995).

## Diagnosis

**Figure 1:**
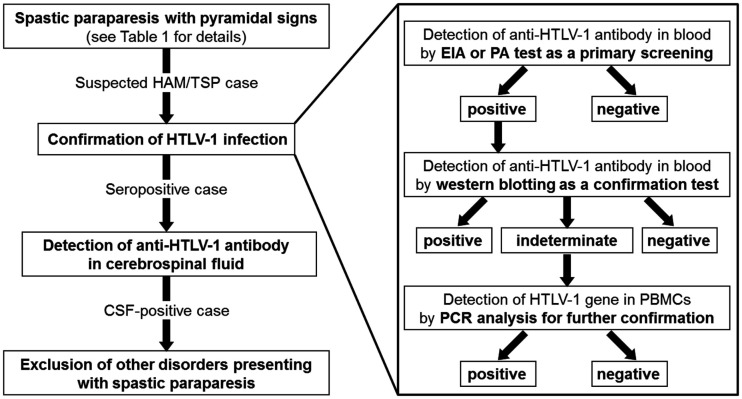
**Flow chart for clinical diagnosis of HAM/TSP**. EIA, enzyme immunoassay; PA, particle agglutination; PBMCs, peripheral blood mononuclear cells; PCR, polymerase chain reaction.

The diagnosis of HAM/TSP is based upon a combination of characteristic clinical features and confirmation of HTLV-1 infection, along with exclusion of other disorders presenting spastic paraparesis (Figure 1). For confirmation of HTLV-1 infection, serological screening for HTLV-1 antibodies can be performed by using a commercially available enzyme immunoassay or particle agglutination test. Confirmatory testing for screening-positive individuals is necessary to eliminate false positives and discriminate between HTLV-1 and HTLV-2. Serological confirmation can be performed by using a commercially available western blot test. Polymerase chain reaction analysis on a blood sample may also be required if the western blot test provides some indeterminate results.

Diagnostic criteria for HAM/TSP were agreed upon by a World Health Organization (WHO) (1989; Table 2). However, a recent recommendation proposes a redefinition of the WHO diagnostic guidelines by formulating levels of ascertainment (definite, probable, and possible), where a patient with definite HAM/TSP manifests non-remitting progressive spastic paraparesis and positive serology and/or detection of proviral DNA, with other disorders being excluded (De Castro-Costa et al., 2006).

**Table 2:** **World Health Organization diagnostic criteria for HAM/TSP**.

Age and sex incidence	Mostly sporadic and adult, but sometimes familial; occasionally seen in childhood; females predominant
Onset	Usually insidious but may be sudden
Main neurological manifestations	Chronic spastic paraparesis, which usually progresses slowly, sometimes remaining static after initial progression
	Weakness of the lower limbs, more marked proximally
	Bladder disturbance usually an early feature; constipation usually occurs later; impotence or decreased libido is common
	Sensory symptoms such as tingling, pins and needles, and burning are more prominent than objective physical signs
	Low lumbar pain with radiation to the legs is common
	Vibration sense is frequently impaired; proprioception is less often affected
	Hyperreflexia of the lower limbs, often with clonus and Babinski’s sign
	Hyperreflexia of the upper limbs, positive Hoffman’s and Tromner signs frequent; weakness may be absent
	Exaggerated jaw jerk in some patients
Less frequent neurological findings	Cerebellar signs, optic atrophy, deafness, nystagmus, other cranial nerve deficits, hand tremor, absent, or decreased ankle jerk. Convulsions, cognitive impairment, dementia, or impaired consciousness are rare
	Muscular atrophy, fasciculations (rare), polymyositis, peripheral neuropathy, polyradiculopathy, cranial neuropathy, meningitis, encephalopathy
Systemic non-neurological manifestations	Pulmonary alveolitis, uveitis, Sjogren’s syndrome, arthropathy, vasculitis, ichthyosis, cryoglobulinemia, monoclonal gammopathy, adult T cell leukemia/lymphoma
Laboratory diagnosis	Presence of HTLV-1 antibodies or antigens in blood and CSF
	CFS may show mild lymphocyte pleiocytosis
	Lobulated lymphocytes may be present in blood and/or CSF
	Mild to moderate increase of protein may present in CSF

*CSF, cerebrospinal fluid*.

Detection of anti-HTLV-1 antibodies in cerebrospinal fluid (CSF) is necessary for the diagnosis of HAM/TSP, based on the WHO diagnostic guidelines. CSF examination revealed mild lymphocyte pleocytosis in approximately one-third of cases as well as mildly elevated protein concentration and increased concentrations of inflammatory markers such as neopterin (Nakagawa et al., 1995; Milagres et al., 2002). These abnormalities can persist for as long as 10 years or more after symptom onset (Moreno-Carvalho et al., 1995).

Spinal cord magnetic resonance imaging (MRI) was abnormal in 3/21 (14%) patients with HAM/TSP in a small series where spinal cord atrophy was reported mainly in the thoracic region (Bagnato et al., 2005). High signal intensity and contrast enhancement with or without associated spinal cord swelling located at cervical or thoracic levels are occasionally observed (Umehara et al., 2007). It has been suggested that patients with more rapidly progressive disease who are scanned earlier in the disease course are more likely to show high signal intensity and contrast enhancement in the spinal cord on MRI, possibly because this reflects highly active spinal cord inflammation.

The differential diagnosis for HAM/TSP includes multiple sclerosis (MS), neuromyelitis optica (NMO), spinal cord compression (e.g., cervical spondylosis and spinal tumors), transverse myelitis, collagen vascular disease, Sjögren syndrome, hereditary spastic paraparesis, primary lateral sclerosis, subacute combined degeneration secondary to vitamin B12 and folate deficiency, human immunodeficiency virus-associated vacuolar myelopathy, neurosyphilis, and Lyme disease, among others. Differentiating, rapidly progressing HAM/TSP from NMO is important. NMO shows more rapid progression than HAM/TSP, and HAM/TSP usually does not present with optic neuritis. Importantly, from our clinical experience, HAM/TSP patients are negative for a specific diagnostic antibody for NMO termed NMO-IgG or anti-aquaporin-4 antibodies (data not published). Furthermore, differentiating HAM/TSP from primary progressive MS is occasionally a diagnostic challenge, since the two conditions are clinically indistinguishable and the mere presence of positive HTLV-1 serology does not necessarily lead to neurological disease. This diagnostic difficulty is compounded by the fact that sometimes, white matter abnormalities are found on brain MRI of HAM/TSP patients (Kira et al., 1991; Alcindor et al., 1992; Kuroda et al., 1995). CSF pleocytosis, when present, typically falls within a similar range, and oligoclonal bands may be present in both. A recent study suggests that a high ratio of proviral DNA load in CSF to peripheral blood mononuclear cells (PBMCs) may distinguish HAM/TSP from HTLV-1-infected patients with MS (Puccioni-Sohler et al., 2007). In general, HTLV-1 proviral loads measured in the CSF of HAM/TSP patients are typically greater than twice the proviral load in PBMCs (Nagai et al., 2001; Takenouchi et al., 2003), whereas the ratio of CSF to peripheral blood HTLV-1 proviral loads are typically lower in asymptomatic carriers (Lezin et al., 2005; Puccioni-Sohler et al., 2007), reflecting either recruitment or expansion of HTLV-1-infected cells in the central nervous system (CNS).

## Pathophysiology

The primary neuropathological feature of HAM/TSP is chronic meningomyelitis of the white and gray matter, followed by axonal degeneration preferentially affecting the middle to lower thoracic cord. Histopathological studies have shown loss of myelin and axons in the lateral columns, with variable damage to anterior and posterior columns in patients with HAM/TSP. The lesions are associated with perivascular and mild parenchymal lymphocytic infiltration with the presence of foamy macrophages, proliferation of astrocytes, and fibrillary gliosis. Later in the course of the disease, the process becomes less cellular and more atrophic. Interestingly, patients who underwent prior steroid treatment show a lesser degree of inflammation (Iwasaki, 1990; Yoshioka et al., 1993; Izumo et al., 2000). Proinflammatory cytokines such as tumor necrosis factor (TNF)-α, interferon (IFN)-γ, and interleukin (IL)-1β were detected in perivascular infiltrating cells (Umehara et al., 1994). There is no direct evidence that HTLV-1 infects neurons, astrocytes, or microglia, but infected CD4^+^ T cells have been observed within spinal cord lesions (Matsuoka et al., 1998), and CD8^+^ T cells directed against HTLV-1 antigens accumulate in the CSF of patients with HAM/TSP (Nagai et al., 2001; Kubota et al., 2002). Immunohistochemical analysis of affected spinal cord lesions in early-stage HAM/TSP patients revealed the presence of infiltrating CD4^+^ and CD8^+^ lymphocytes, among which CD8^+^ cells become increasingly dominant over the duration of the illness (Umehara et al., 1993). The expression of HLA class I antigens has also been found in such lesions (Moore et al., 1989). In addition, infiltrating CD8^+^ CTLs were positive for TIA-1, a CTL marker (Umehara et al., 1994). The number of TIA-1^+^ cells was clearly related to the amount of proviral DNA *in situ*, and the number of infiltrating CD8^+^ cells appeared to correlate with the presence of apoptotic cells.

Human T-lymphotropic virus type 1-1-infected CD4+ T cells may primarily contribute to development of HAM/TSP, since the number of HTLV-1-infected T cells circulating in the peripheral blood is higher in patients with HAM/TSP than in asymptomatic HTLV-1-infected individuals (Nagai et al., 1998; Yamano et al., 2002); this number is even higher in the CSF of patients with HAM/TSP (Nagai et al., 2001). Recently, CD4^+^CD25^+^CCR4^+^ T cells, which mainly include suppressive T cell subsets such as regulatory T (T_reg_) cells under healthy conditions, are the predominant viral reservoir of HTLV-1 in both ATLL and HAM/TSP (Yoshie et al., 2002; Yamano et al., 2009). Interestingly, cells of this T cell subset become Th1-like cells with overproduction of IFN-γ in HAM/TSP, while in ATLL patients, leukemogenesis develops, and maintains the T_reg_ phenotype. These results indicate that HTLV-1-infected T cells are increased and abnormally modified, favoring the development of HAM/TSP.

Human T-lymphotropic virus type 1-associated myelopathy/tropical spastic paraparesis patients show extremely high cellular and humoral acquired immune responses, such as high frequencies of Tax-specific CD8^+^ T cells in peripheral blood and CSF (Jacobson et al., 1990; Nagai et al., 2001); high antibody titer to HTLV-1 (Ishihara et al., 1994; Akahata et al., 2012); and increased production of proinflammatory cytokines such as IL-6, IL-12, and IFN-γ (Furuya et al., 1999). Recently, overexpression of a subset of IFN-stimulated genes in HAM/TSP patients was demonstrated using systems biology approaches (Tattermusch et al., 2012).

While the acquired immune response is accelerated, HAM/TSP patients demonstrate reductions in the amount and efficacy of cellular components of innate immunity; this is vital for regulating the immune response against general viral infections and cancers. The numbers and functions of CD56^+^CD16^+^ natural killer (NK) cells in HAM/TSP patients are significantly lower than those observed in healthy controls (Yu et al., 1991; Azakami et al., 2009). In addition, HAM/TSP patients also have a decreased frequency of invariant natural killer T (iNKT) cells in peripheral blood (Azakami et al., 2009; Ndhlovu et al., 2009).

**Figure 2:**
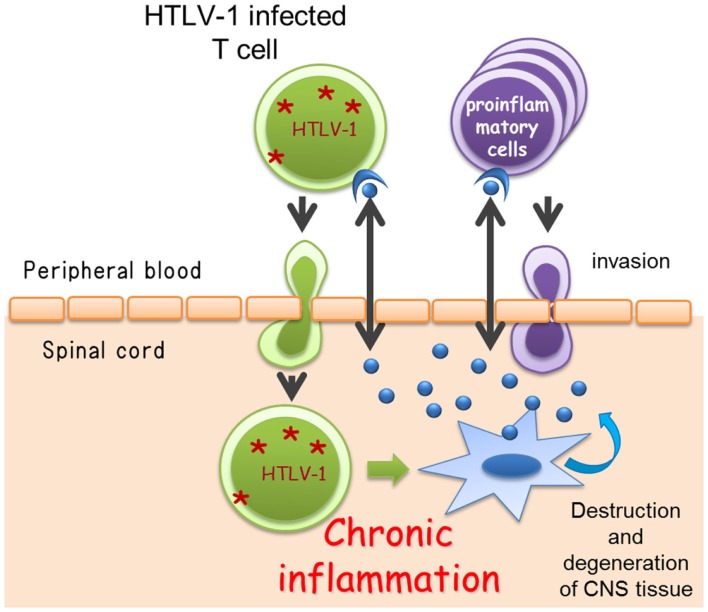
**Cellular mechanisms underlying pathogenesis of human T-lymphotropic virus type 1-associated myelopathy/tropical spastic paraparesis (HAM/TSP)**. CNS, central nervous system. *HTLV-1.

Although the exact cellular and molecular events underlying the induction of chronic inflammation in the spinal cord by HTLV-1 are still unclear, the most widely accepted hypothesis is that HAM/TSP is the result of “bystander damage” (Ijichi et al., 1993; Nagai et al., 2001; Osame, 2002). The sequence of events leading to bystander damage may be as follows. Activation of HTLV-1-infected CD4^+^ T cells induce high-migration activity (Furuya et al., 1997; Kambara et al., 2002) and allows the migration of infected CD4^+^ T cells across the blood–brain barrier from the peripheral blood to the CNS. Migrated HTLV-1-infected CD4^+^ T cells start to express viral antigens, including Tax, and secrete proinflammatory cytokines such as IFN-γ (Hanon et al., 2001; Kambara et al., 2002), which stimulate the resident cells to produce multiple chemokines. These chemokines recruit more proinflammatory cells including HTLV-1-infected CD4^+^ T cells and HTLV-1-specific CD8^+^ T cells that are preferentially recruited and/or expanded in the CNS. Thus, HTLV-1-specific immune responses and secondary inflammations inflated in the CNS may lead to the subsequent CNS damage (Figure 2).

## Prognosis

**Figure 3:**
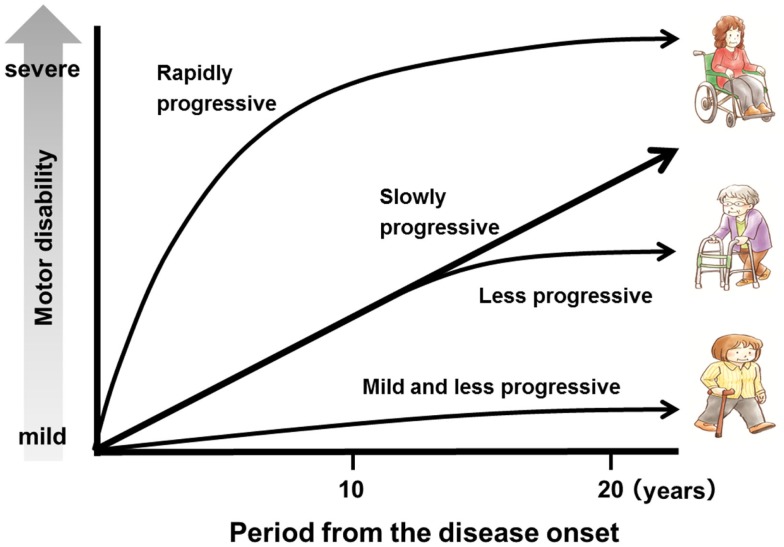
**A schematic representation of the clinical course of human T-lymphotropic virus type 1-associated myelopathy/tropical spastic paraparesis (HAM/TSP)**.

The symptoms usually begin during adulthood, most frequently after the age of 40 years (range, 6–75 years). The disease usually progresses slowly without remission. However, there is a subgroup of patients with rapid progression who are unable to walk within 2 years, and another subgroup of patients with very mild progression (Nakagawa et al., 1995; Gotuzzo et al., 2004; Olindo et al., 2006; Lima et al., 2007; Martin et al., 2010). Indeed, in HAM/TSP, the clinical course and rate of progression may vary greatly among patients (Figure 3). In a study of 123 patients with a 14-year follow-up, the median time from symptom onset to need for unilateral walking aid was 6 years; bilateral walking, 13 years; and wheelchair dependence, 21 years. Nineteen of those 123 patients died due to complications of HAM/TSP, and the mean age at death was approximately 15 years shorter than the life expectancy in the cohort area (Olindo et al., 2006). In a study of 48 patients with a 15-year follow-up, the median time from symptom onset to the need for unilateral walking aid was 11 years; bilateral walking, 11.2 years; and wheelchair dependence, 18 years. The conditions of 3 of these 48 patients worsened rapidly, and they were unable to walk within 2 years, while in six patients, the progression was slow or the condition did not worsen; 5 of the 48 patients died, and the median age at death was 57 years (range, 36–78 years). Importantly, a timed 10-m walk was found to be a more sensitive scale to identify motion deterioration and recognize patients in need of therapeutic intervention (Martin et al., 2010). In terms of vital prognosis, it is also important to recognize that HAM/TSP patients have a risk to develop ATLL (Tamiya et al., 1995).

Since HAM/TSP is a chronic progressive neurological disease, the progression of clinical disease is usually subtle; this hampers the evaluation of disease progression even over the course of a year. Therefore, information about quantitative biomarkers associated with disease prognosis and disease activity is important for assessing the effect of therapy as well as conducting clinical trials of novel therapeutics with statistically significant endpoints. Although few well-designed studies have evaluated the usefulness of potential biomarkers as surrogate markers, accumulating evidence supports the relationship between HTLV-1 proviral load and long-term disease prognosis. Indeed, in a study with 100 untreated HAM/TSP patients, a significant association was demonstrated between higher HTLV-1 proviral load and poor long-term prognosis (Olindo et al., 2005); later, the authors confirmed this result in a bigger cohort (Olindo et al., 2006). Analysis of observational studies also showed a relationship between HTLV-1 proviral load and disease prognosis (Matsuzaki et al., 2001; Takenouchi et al., 2003). Older age at onset has also been demonstrated to be associated with poor long-term prognosis (Nakagawa et al., 1995; Matsuzaki et al., 2001; Olindo et al., 2006). In terms of biomarkers of disease activity, recent work by our research group showed that CSF cell count, neopterin concentration, and CSF levels of C-X-C motif chemokine 10 are well correlated with disease progression over 4 years, better even than HTLV-1 proviral load in PBMCs (manuscript in preparation). A prospective study to determine whether these indicators are useful as prognostic biomarkers will be necessary.

## Treatment

Since the discovery of HAM/TSP, various therapeutic approaches have been used for HAM/TSP patients. However, no effective therapeutic strategy has been established thus far. Because induction of chronic inflammation by HTLV-1-infected T cells in the spinal cord is considered the major pathogenic mechanism underlying HAM/TSP, anti-inflammatory, or antiviral therapies have been tested. Clinical improvements in open-label studies have been reported for a number of agents including corticosteroids (Nakagawa et al., 1996), danazol (Harrington Jr. et al., 1991), pentoxifylline (Shirabe et al., 1997), and IFN-β1 (Oh et al., 2005). With the exception of IFN-α (Izumo et al., 1996), however, these drugs lack evidence required to merit strong recommendation for their use in HAM/TSP. The role of IFN-α in HAM/TSP is also not clear, as no study has conclusively shown its long-term benefit. Here, I summarize the results of recent trials and discuss the need for the identification of novel drug targets (Table 3).

**Table 3:** **Summary of reports on treatment for HAM/TSP**.

Authors	Country	Study design	Reagents	Treatment regimen	Studyperiod	No. of patients	Rate of Efficacy	Note
Osame et al. (1990b)	Japan	Open-label	Prednisolone	60–80 mg qod for 2 month	11 Month	65	90.8% (59/65)	Incidence of side effects: 20% (13/65)
				→10 mg off/month for 6 month			56.9% (>1)	
				→5 mg qod for 3 month	
Croda et al. (2008)	Brazil	Case series	Methylprednisolone	1 g × 3 days/month for 3–4 month	2.2 Years	39	24.5%	Transient effect
Nakagawa et al. (1996)	Japan	Open-label	Prednisolone	1–2 mg/kg qd or qod for 1–2 month → tapering	6–12 Month	131	81.7%	Decrease of CSF neopterin
							69.5% (>1)	
			Methylprednisolone	500 mg–1g × 3 days		10	30.0%	For rapid progression
			Interferon-α	3 MU/day × 30 days	1–3 Month	32	62.5%	Transient effect
							21.9% (>1)	Incidence of side effects: 65.6% (21/32)
Martin et al. (2012)	UK	Open-label	Cyclosporine A	2.5–5 mg/kg/day bd for 48 week	72 Week	7	71.4% (5/7) after 3 Month	Clinical failure: two patients
Izumo et al. (1996)	Japan	Multicenter double-blind RCT	Interferon-α	0.3 MU/day × 28 days	8 Week	15	7.1%	Incidence of side effects: 26.7% (4/15)
				1 MU/day × 28 days		17	23.5%	29.4% (5/17)
				3 MU/day × 28 days		16	66.7%	50.0% (8/16)
Yamasaki et al. (1997)	Japan	Case series	Interferon-α	6 MU/day × 14 days → 6 MU/3 times/week × 22 week	6 Month	7	71.4% (5/7)	Clinical failure: two patients
Arimura et al. (2007)	Japan	Phase IV	Interferon-α	3 MU/day × 4–793 days (median 30 days)	6 Month	167	66.2%	Side effects: 87.4%
							29.2% (>1)	Serious side effects: 7.0%
Taylor et al. (2006)	UK and Japan	Double-blind RCT	Zidovudine + lamivudine	AZT 300 mg + 3TC 150 mg bd	48 Week	16	No clinical improvement	No change in proviral load
Macchi et al. (2011)	UK	Case series	Tenofovir	245 mg/day	2–16 Month	6	No clinical improvement	No change in proviral load

*>1, improvement of more than one grade in the Osame’s motor disability score*.

*No., number; qod, every other day; mo: month(s); yr, year(s); qd, every day; MU, million unit; wk, week(s)’ bd, twice daily; RCT, randomized controlled trial; AZT, zidovudine; 3TC, lamivudine*.

Soon after the definition of HAM/TSP, corticosteroids were reported to decelerate the progression of this disease (Osame et al., 1990b). In a large-scale case series study (Nakagawa et al., 1996), oral prednisolone was effective in 81.7% of 131 patients, with 69.5% of the 131 patients showing more than one grade of improvement, as determined by Osame’s motor disability scale. Furthermore, oral prednisolone therapy decreased the concentration of neopterin, which is an inflammatory marker of HAM/TSP, in CSF (Nakagawa et al., 1996). A recent open-cohort study of 39 patients with HAM/TSP with a mean follow-up of 2.2 years showed an improvement in overall disability following pulsed intravenous methylprednisolone (Croda et al., 2008). However, a few studies reported no such benefit (Kira et al., 1991; Araújo et al., 1993), and there has been no randomized clinical trial. Although steroidal therapy is not recognized as a radical therapy since it does not eliminate the HTLV-1-infected cells, in practice, steroids are the most commonly prescribed drug, despite the poor evidence for their efficacy. This is probably because some patients experience highly active inflammation or there is a significant inflammatory phase relatively early in disease. Since the clinical course and disease activity of HAM/TSP vary among patients (Figure 3), the treatment plan should be designed based on the patient’s background such as activity or phase of the disease.

It is also notable that some patients’ condition worsened after the dose of prednisolone was reduced, and hence, these patients remain dependent on drug administration (Nakagawa et al., 1996). In my research group, we had similar experiences; we found that such patients usually have high inflammatory levels in CSF, which increase even more as the clinical situation worsens after the dose of prednisolone is decreased. Since long-term use of prednisolone therapy is not desirable due to its variety of side effects, the development of steroid-sparing agents is urgently required for these patients. Candidate steroid-sparing agents could be anti-inflammatory and/or antiviral in nature. In fact, there is a recent report on the high efficacy of cyclosporine A therapy targeted at early phase or progressive HAM/TSP patients. In this study, clinical improvement was observed in five of seven patients, with reduction of provirus DNA load observed in the CSF (Martin et al., 2012).

Type I IFNs (α and β), which have immunomodulatory and antiviral properties (Borden et al., 2007), have been tested as anti-HAM/TSP drugs. IFN-α demonstrated clinical benefits in a multicenter, randomized, double-blind, controlled trial of HAM/TSP patients in Japan (Izumo et al., 1996). In this study, 3 million units (MU) of human lymphoblastoid natural IFN-α given daily by intramuscular injection for 28 days showed better clinical benefit than 0.3 or 1 MU of IFN-α. The reduction of proviral DNA load and memory CD8^+^ cells in PBMCs (Saito et al., 2004) and the reduction of CD4/CD8 ratio and CD4^+^CCR5^+^ cells in CSF (Kambara et al., 2002) after short-term IFN-α therapy was demonstrated. However, the benefit of long-term IFN-α therapy has not been well demonstrated. A small study extending IFN-α treatment for 24 weeks reported sustained clinical response (Yamasaki et al., 1997). In a post-marketing surveillance of IFN-α in Japan, sustained improvements in motor disability for 5 months after cessation of IFN-α administration were observed in 11 of 30 patients, and a high adverse event rate (536 events reported in 146 patients; 46 classified as serious) was indicated (Arimura et al., 2007). In this surveillance study, it is notable that IFN-α treatment was more effective in patients with lower motor disability and shorter duration of illness and progression phase, suggesting the existence of therapeutic windows of opportunity in the treatment of HAM/TSP. It is also notable that rapidly progressing HAM/TSP patients showed no response and dropped out from the IFN-α therapy (Yamasaki et al., 1997; Arimura et al., 2007). Therefore, well-designed controlled clinical trials to guide the clinician with regard to the appropriate target, time of initiation, and the dose or duration of IFN-α therapy in HAM/TSP will be important for future studies.

Thus, corticosteroids and IFN-α may have therapeutic efficacy for HAM/TSP to some extent; however, the effect may not be sufficient for avoiding long-term disability. Moreover, in some cases, it might be difficult to continue therapy because of the side effects of these drugs and their insufficient benefit. Therefore, it is essential that revolutionary drugs that can lead to a paradigm shift in the therapeutic strategies for HAM/TSP be developed. Considering the pathogenesis of HAM/TSP, therapies to eliminate HTLV-1-infected cells from the peripheral blood and CNS should be developed. However, antiviral therapy has not been successful in the clinical trial for HAM/TSP. A randomized, double-blind, placebo-controlled, 6-month study of zidovudine and lamivudine combination therapy, which demonstrated activity against HTLV-1 reverse transcriptase *in vitro*, was conducted in 16 patients, and no significant changes were observed in the clinical symptoms and HTLV-1 proviral load (Taylor et al., 2006). A pilot trial of tenofovir, which also demonstrated activity against HTLV-1 reverse transcriptase *in vitro*, was conducted in six patients, and no significant change in HTLV-1 proviral load was observed (Macchi et al., 2011). Thus, the impact of therapy with viral reverse transcriptase inhibitors with the aim of reducing the HTLV-1 proviral load *in vivo* has been minimal. These results support the hypothesis that HTLV-1 proviral load in HTLV-1-infected patients is mainly maintained through cell division of infected cells and not by viral replication and new infection (Wattel et al., 1995; Cavrois et al., 1998). Therefore, development of therapies directly targeting HTLV-1-infected cells could be more promising to reduce the viral load. Recently, we have demonstrated that CC chemokine receptor 4 (CCR4), expressed on the surface of ATLL cells (Ishida et al., 2004), is also expressed on HTLV-1-infected cells in HAM/TSP patients (Yamano et al., 2009). More recently, a humanized anti-CCR4 monoclonal antibody has been developed; the safety and efficacy of this antibody has been proven in phase I and II studies (Yamamoto et al., 2010; Ishida et al., 2012) and subsequently approved by the Ministry of Health, Labour and Welfare as a therapeutic agent for relapsed patients with ATLL in Japan. Further clinical trials on the safety and efficacy of anti-CCR4 therapy for HAM/TSP patients should be conducted in future studies.

## Conclusion

Advances in study of the epidemiology and pathogenesis of HAM/TSP have led to the identification of several biomarkers and therapeutic targets. However, these findings have not yet translated into an optimal therapeutic strategy for this hitherto intractable neurological disease. Well-designed clinical trials in HAM/TSP will provide opportunities for further quantification of biomarkers and refinement of therapeutic drugs. The development of an effective therapy to improve long-term prognosis in HAM/TSP is of paramount importance, and clinical trials for the validation of HAM/TSP relevant biomarkers and new therapeutic targets will be key challenges in this therapy.

## Conflict of Interest Statement

The authors declare that the research was conducted in the absence of any commercial or financial relationships that could be construed as a potential conflict of interest.
